# A Novel *FLCN* Intragenic Deletion Identified by NGS in a BHDS Family and Literature Review

**DOI:** 10.3389/fgene.2021.636900

**Published:** 2021-04-01

**Authors:** Minghui Cai, Xinxin Zhang, Lizhen Fan, Shuwen Cheng, Abdukahar Kiram, Shaoqin Cen, Baofu Chen, Minhua Ye, Qian Gao, Chengchu Zhu, Long Yi, Dehua Ma

**Affiliations:** ^1^Department of Cardiothoracic Surgery, Taizhou Hospital of Zhejiang Province affiliated to Wenzhou Medical University, Linhai, China; ^2^Department of Histology and Embryology, School of Medicine, Southeast University, Nanjing, China; ^3^Jiangsu Key Laboratory of Molecular Medicine, School of Medicine, Nanjing University, Nanjing, China

**Keywords:** BHD syndrome, *FLCN* gene, large intragenic deletion, targeted NGS, MLPA

## Abstract

Birt–Hogg–Dubé syndrome (BHDS, MIM #135150), caused by germline mutations of *FLCN* gene, is a rare autosomal dominant inherited disorder characterized by skin fibrofolliculomas, renal cancer, pulmonary cysts and spontaneous pneumothorax. The syndrome is considered to be under-diagnosed due to variable and atypical manifestations. Herein we present a BHDS family. Targeted next generation sequencing (NGS) and multiplex ligation-dependent probe amplification (MLPA) revealed a novel *FLCN* intragenic deletion spanning exons 10-14 in four members including the proband with pulmonary cysts and spontaneous pneumothorax, one member with suspicious skin lesions and a few pulmonary cysts, as well as two asymptomatic family members. In addition, a linkage analysis further demonstrated one member with pulmonary bullae to be a BHDS-ruled-out case, whose bullae presented more likely as an aspect of paraseptal emphysema. Furthermore, the targeted NGS and MLPA data including our previous and present findings were reviewed and analyzed to compare the advantages and disadvantages of the two methods, and a brief review of the relevant literature is included. Considering the capability of the targeted NGS method to detect large intragenic deletions as well as determining deletion junctions, and the occasional false positives of MLPA, we highly recommend targeted NGS to be used for clinical molecular diagnosis in suspected BHDS patients.

## Introduction

Birt–Hogg–Dubé syndrome (BHDS, MIM #135150), caused by germline mutations of *FLCN* gene, is a rare autosomal dominant inherited disorder characterized by skin fibrofolliculomas, renal cancer, pulmonary cysts and spontaneous pneumothorax (Roth et al., 1993; Birt et al., 1977; Nickerson et al., 2002). It is often considered to be underdiagnosed due to variable and atypical manifestations. Multiple and bilateral pulmonary cysts are the most common manifestation of BHDS and can be observed in more than 80% of BHDS patients (Zbar et al., 2002; Schmidt et al., 2005; Toro et al., 2007; Agarwal et al., 2011). They could exhibit a pneumothorax dominant phenotype with no or reduced penetrance of the skin or renal manifestations (Ren et al., 2008). It is in some degree challenging for clinicians or radiologists to distinguish these BHDS patients from patients with other cystic lung diseases such as lymphangioleiomyomatosis, lymphoid interstitial pneumonia and Langerhans cell histiocytosis (Raoof et al., 2016). However, various cystic lung diseases could have a characteristic CT appearance in terms of distribution, extent and morphology of cysts (Agarwal et al., 2011; Raoof et al., 2016), that allows their distinction, which could narrow the differential diagnosis considerably. Early and accurate diagnosis of BHDS is crucial, which will lead to early identification and treatment of renal cancer in patients and their family members. Due to the variability in the clinical manifestations and the complicacy of diagnostic criteria (Menko et al., 2009), a DNA-based diagnosis is necessary.

*FLCN*, currently the only gene known to be associated with BHDS, is located on chromosome 17p11.2, consists 14 exons, and encodes an evolutionarily conserved protein whose function has not yet been completely understood. As is shown in the online Locus-Specific Database for *FLCN*^1^ (Lim et al., 2010), there are 286 unique public DNA variants (last accessed: 08/31/2020), in which small indels and nonsense mutations account for the majority of pathogenic variants detected by DNA sequencing. Whereas, splice-site mutation and large intragenic deletions/duplications are also vital because these pathogenic mutation can lead to premature protein truncation or haploinsufficiency (Kunogi et al., 2010; Furuya et al., 2016; Xu et al., 2016; Jensen et al., 2017).

At present, DNA-based diagnosis of BHDS mainly relies on Sanger sequencing and multiplex ligation-dependent probe amplification (MLPA), which makes the diagnosis more time-consuming and labor-intensive. Additionally, there are different kinds of complex mutations in *FLCN* that may not be detected by Sanger sequencing, such as large intragenic deletions or duplications and deep intronic mutations that may lead to abnormal splicing (Benhammou et al., 2011; Ding et al., 2015). Therefore, a new rapid next generation sequencing (NGS) strategy which could identify not only point mutations and indels but also copy number variations (CNV) in *FLCN* gene was developed to conduct the molecular diagnosis of BHDS (Zhang et al., 2016).

In the current study, six individuals including three patients with primary spontaneous pneumothorax (PSP) or lung cysts/bullae from a three-generation family were investigated. Linkage analysis and targeted NGS were both conducted to identify the potential mutation of *FLCN* gene.

## Case Presentation

### Clinical Report

**FIGURE 1:**
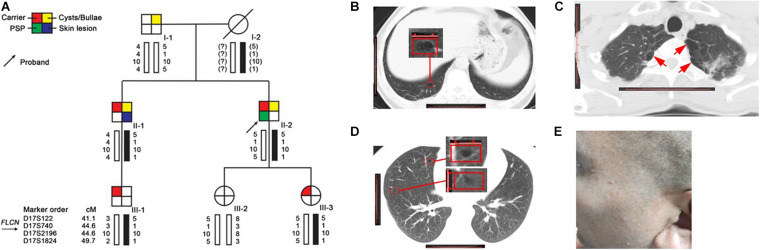
Pedigree and clinical pictures of the family. **(A)** Pedigree and haplotype analysis. Numbers shown below each individual indicate the genotypes of the microsatellite markers in chromosomal order. The black bars represent the disease-associated haplotype. **(B–D)** Representative CT images of II-2 **(B)**, I-1 **(C)**, and II-1 **(D)**; red frame: cyst; red arrow: bullae. **(E)** Several dome-shaped, skin-colored papules were seen on the face of II-1.

The family (Figure 1A) was ascertained through a proband (II-2) with spontaneous pneumothorax, he came to the Taizhou Hospital at the age of 44 for his second attack, who had his first episode at age of 43. He was a non-smoker and he had neither skin lesion nor renal cancer. The chest computed tomography (CT) showed that he had a right-sided pneumothorax and multiple bilateral pulmonary cysts in different pulmonary segments, especially the basal lung region (Figure 1B). The CT scan was performed in each family member except the proband’s mother (I-2) who died of colon cancer 20 years ago and revealed that the proband’s father (I-1, 71 years old) and brother (II-1, 40 years old) had cysts or bullae (Figures 1C,D), the others (III-1, 24 years old, III-2, 14 years old and III-3, 8 years old) had no obvious abnormity in the lung. The CT images of I-1 who was a heavy smoker and had never suffered from spontaneous pneumothorax showed polygonal-shaped, emphysematous subpleural bullae in the upper lobe (Figure 1C). The CT images of II-1, who had skin lesions on his face and neck with multiple dome-shaped, white or skin-colored papules (Figure 1E), showed that he had a few 5–8 mm thin-walled cysts in the upper and middle lobes (Figure 1D).

### Genetic Analysis

**FIGURE 2:**
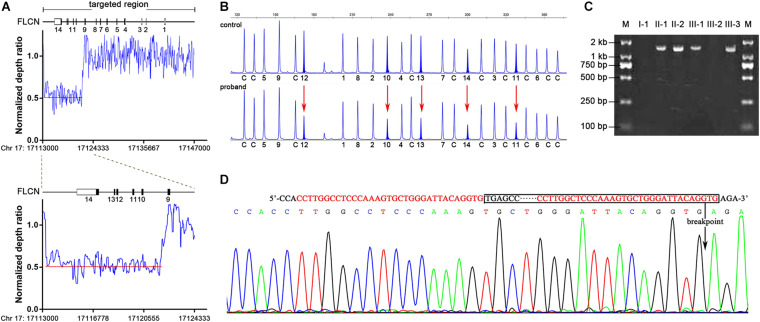
Identification of *FLCN* intragenic deletions and breakpoint analysis in II-2. **(A)** Representation of the normalize depth ratio of 20-bp intervals in *FLCN* gene detected by NGS method. The red dotted lines represent the average ratio of the deletion area. **(B)** MLPA analysis showed heterozygous exonic deletions (red arrows) in II-2 compared with the control individual. The characters and numbers below the probes indicate exons and control probes. **(C)** PCR products of junction fragments were separated by 2% agarose gel electrophoresis. The numbers above the lanes corresponded to the subjects of the Pedigree in Figure 1A. **(D)** Bidirectional sequencing of the junction region. The sequences of microhomology between the up and downstream breakpoints were marked in red; boxed nucleotides indicate the deletion and an arrow indicate the location of the deletion.

Mutation analysis of *FLCN* was performed and no pathogenic point or indel mutation was identified by Sanger sequence analysis. As phenotype analysis in this family could not exclude the possibility of BHDS, we performed a targeted NGS in twelve suspect BHDS patients without any pathogenic mutation of *FLCN* gene including the proband. Comprehensive analysis including CNV analysis was subsequently carried out. By introducing a normalized depth-based method (Zhang et al., 2016), we detected a novel large deletion spanning exons 10–14 in the proband (Supplementary Figure 1 and Supplementary Table 3), who was diagnosed as BHDS consequently. By calculating the normalized depth of a set of 20 bp-interval reads covered the entire *FLCN* gene, we defined the approximate sites of the breakpoints (Figure 2A). One breakpoint is located near chr 17: 17113680, the other breakpoint is near chr 17: 17121880, the estimated deletion size is approximately 8.2 kb in length.

Multiplex ligation-dependent probe amplification assay was subsequently performed to validate and determine the large deletion in proband and family members mentioned above. The large deletion of exons 10–14 in the proband detected by NGS method was confirmed (Figure 2B), and MLPA analysis showed that the large deletion also occurred in II-1, III-1 and III-3.

To determine the precise breakpoint, the junction fragments adjacent to the deleted regions were amplified by Polymerase Chain Reaction (PCR) with specially designed primers. The 1.5 kb PCR product of the junction fragments were separated by agarose gel electrophoresis, while the 10.0 kb wild-type sequences were too long to be amplified (Figure 2C). Bidirectional sequencing of the PCR products indicated an 8,384 bp deletion encompassing exons 10–14 (chr17: 17113559-17121942) (Figure 2D), the mutation was not recorded in the online Locus-Specific Database for *FLCN*^2^ or ClinVar^3^. Further analysis illustrated that the up- and down-stream breakpoints of the deletion were flanked by Alu-Sg and Alu-Sx1 repeats, respectively, in which there were microhomology-mediated break-induced replication sequences (Figure 2D).

As results showed that I-1, who also exhibited multiple pulmonary bullae, did not carry the mutation as others (II-1, II-2, III-1, and III-3). We conducted a linkage analysis to validate the segregation of the mutation. Evidence of linkage was observed in family members with the large deletion mutation, and inferred that the father (I-1), did not pass on the affected haplotype (5-1-10-1) to the two affected children and this has ruled him out as the obligate carrier. The affected haplotype was transmitted from the mother (I-2) (Figure 1A), suggesting that I-2 should be regarded as an obligate carrier.

**FIGURE 3:**
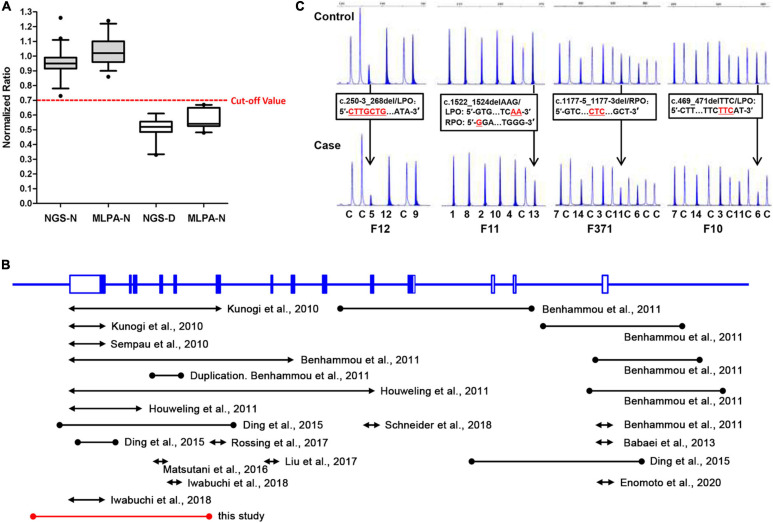
A comparative analysis of NGS and MLPA and a review of previously reported *FLCN* intragenic deletions. **(A)** Box-plot (median, box: 5–95th percentile, whiskers) representation of haploid copy number values for *FLCN* deleted exons (NGS-D and MLPA-D) and normal controls (NGS-N and MLPA-N) by NGS and MLPA, respectively. The red dotted line represents the cut-off value; exons with values <0.7 were scored as deleted, while >0.7 as normal status. **(B)** Genomic structure of the *FLCN* gene, showing the locations and sizes of previously reported large intragenic deletions found in BHD patients; black line: deletions previously reported. Red line: deletion found in this study. The lines with solid circles or arrowheads at both ends indicate the deletions with breakpoints determined or undetermined, respectively. **(C)** Small indels can show peak heights of heterozygous exonic deletions (normalized ratio <0.7) compared with the control individuals (black arrow) in MLPA analysis. DNA changes/*FLCN* probe sequences are presented in black frames, respectively. LPO (Left Probe Oligo) is the 5′ half of the probe; RPO (Right Probe Oligo) is the 3′ half of the probe. Red underlined characters indicate deleted bases. The characters and numbers below the probes indicate exons and control probes. The number below each MLPA result indicate sample number in our private sample database.

Furthermore, to evaluate the precision and accuracy of the MLPA and NGS approach for detecting exon deletions, we compared the normalized ratio and their standard deviations (SD) for the deleted and non-deleted exons from the present and previous studies (Ding et al., 2015; Zhang et al., 2016). For normal exons, the mean normalized ratio in NGS and MLPA method was 0.9488 (*SD*: 0.0956) and 1.04 (*SD*: 0.1023), respectively. For deleted exons, the mean normalized ratio in NGS and MLPA method was 0.5105 (*SD*: 0.0762) and 0.569 (*SD*: 0.0614), respectively. The corresponding histogram showed that the effectiveness in detecting exon deletions of the two methods is equivalent (Figure 3A). No overlap was seen between the diploid and haploid copy number values in both MLPA and NGS group. We found a cut-off value of 0.7 for scoring a deletion (<0.7) or normal copy number (>0.7) status for both methods.

Detailed methods and a visualized flow chart of deletion detection and precise breakpoints determination (Supplementary Figure 2) were available in the Supplementary Material.

## Discussion and Conclusion

Here we report a novel *FLCN* intragenic deletion spanning exons 10–14 segregated in a BHDS family. In this family, the deletion was detected in two affected members (II-1 and II-2) as well as two asymptomatic carriers (Figure 1A), who were subsequently diagnosed with BHDS. The intragenic deletion spanning exon 10–14 was predicted to result in C-terminal truncation of FLCN (Ding et al., 2015). As previously reported, FLCN interacts with FNIP1 or FNIP2 through its C-terminus, truncating mutations result in loss of the C-terminal region of FLCN, abolish its interaction with FNIP1 and FNIP2, and consequently disrupt its normal function (Baba et al., 2006; Hasumi et al., 2008; Takagi et al., 2008; Woodford et al., 2016).

In this family we reported, for the proband, chest CT scanning reveals multiple bilaterally parenchymal lung cysts predominantly in the basal and periphery lung region (subpleural) (Figure 1B), which is a typical BHDS manifestation (Graham et al., 2005; Agarwal et al., 2011; Raoof et al., 2016). For II-1, despite his atypical CT manifestation, multiple, dome-shaped, whitish or skin-colored papules can be observed on his face and neck, the phenotype is highly considered as BHDS. Both patients are confirmed to be BHDS by genetic testing. However, CT scanning also reveals the pulmonary bullous changes in I-1 (Figure 1C), we found that the location and characteristics of CT feature is more likely an emphysematous destruction (Koo and Yoo, 2013), which is considered mainly related to cigarette smoking. He is eventually determined to be a BHDS-ruled-out case by genetic analysis.

Although chest CT scanning is essential for differential diagnosis, for suspected BHDS patients, we highlight the need for pedigree investigation. Segregation analysis combined with clinical and radiographic features may help differentiate among various cystic lung diseases, and genetic screening is imperative to make a definite diagnosis (Painter et al., 2005).

*FLCN*, responsible for BHDS, is a tumor suppressor gene that was firstly reported in 2002 (Nickerson et al., 2002) and has variable mutation types, most of which are small indels and nonsense mutations detected by Sanger sequencing, and there are also approximately 10% large intragenic deletions and duplications normally detected by MLPA.

Up to now, including our previous and present findings, totally 30 cases or families harboring 24 unique *FLCN* intragenic deletions/duplications have now been reported worldwide (Figure 3B and Table 1) (Kunogi et al., 2010; Sempau et al., 2010; Benhammou et al., 2011; Houweling et al., 2011; Babaei Jandaghi et al., 2013; Ding et al., 2015; Matsutani et al., 2016; Liu et al., 2017; Rossing et al., 2017; Iwabuchi et al., 2018; Schneider et al., 2018; Enomoto et al., 2020). Patients with *FLCN* deletions/duplications exhibit a high degree of interfamilial clinical variability, while no particular phenotype was seen more frequently in association with intragenic deletions (*p* > 0.05) (Supplementary Table 4). The main detection methods were quantitative PCR (qPCR) and MLPA, array-based comparative genomic hybridization (aCGH) was also employed in order to more finely map the deletions (Benhammou et al., 2011). Among the deletions, nine pairs of the breakpoints were determined by long range PCR, six pairs of the deletion breakpoints were flanked by Alu repeats, and one was partially Alu-mediated.

**TABLE 1:** Previous and present reported *FLCN* intragenic deletions/duplications.

	**References**	**cDNA change**	**Deleted exon(s)**	**Detection method**	**Breakpoint detected (yes/no)**	**Deletion size (bp)**	**Clinical manifestations**
(1)	Kunogi et al., 2010	c.872−?_1740+?del	Exon 9–14 del	qPCR	No	–	PTX
		c.1539−?_c.1740+?del	Exon 14 del	qPCR	No	–	PTX
(2)	Sempau et al., 2010	c.1539−?_c.1740+?del	Exon 14 del	MLPA	No	–	PTX, FF, RCC
(3)	Benhammou et al., 2011	c. −227–853_c.397–295del	Exons 2–5 del	qPCR, MLPA, aCGH	Yes, flanked by AluSq and AluSx	9,189	FF, LC
		c.619−?_c.1740+?del	Exons 7–14 del	qPCR, MLPA, aCGH	No	–	FF
		c.−4174_−227–1566del	Exon 1 del	qPCR, MLPA, aCGH	Yes, flanked by AluY and AluSx	6,391	Perifollicular fibroma, RCC
		c.−5575_−228+341delins CCCCCATGG	Exon 1 del	qPCR, MLPA, aCGH	Yes, not flanked by Alu	5,688	FF, LC
		c.−6544_−228+454delins −3779_−3655inv	Exon 1 del	qPCR, MLPA, aCGH	Yes, partially Alu-mediated, AluY and AluSg	6,645	FF, LC
		c. −?_−227−?del	Exon 1 del	qPCR, MLPA, aCGH	No	–	FF, LC, PTX
		c.1063–154_1300+410dup	Exons 10–11 dup	qPCR, MLPA, aCGH	Yes, not flanked by Alu	1,341	FF, LC, RCC
(4)	Houweling et al., 2011	c.250−?_c.1740+?del	Exons 5–14 del	MLPA	No	–	PTX
		c.1301−?_c.1740+?del	Exons 12–14 del	MLPA	No	–	PTX
(5)	Babaei Jandaghi et al., 2013	c. −?_−227−?del	Exon 1 del	qPCR	No	–	PTX, RCC
(6)	Ding et al., 2015	c. −504−1303_−25+845del	Exons 1–3 del	MLPA	Yes, flanked by Alu-Sc and Alu-Sz	7,543	PTX, LC, SL
		c.872−429_1740+1763del	Exons 9–14 del	MLPA	Yes, flanked by Alu-Sx3 and Alu-Sc8	7,747	PTX, LC, SL
		c.1539–536_1740+1071del	Exon 14 del	MLPA	Yes, mediated by 2 Alu-Sq2s with 86.1% identity	1,809	PTX, LC, SL
(7)	Rossing et al., 2017	c.872−?_1062 + ?del	Exon 9 del	MLPA	No	–	LC, RCC
(8)	Matsutani et al., 2016	c.1177−?_1300+?del	Exon 11 del	Not mentioned	No	–	PTX, LC, SL
(9)	Liu et al., 2017	c.780−?_871+?del	Exon 8 del	MLPA	No	–	LC, renal cysts
(10)	Schneider et al., 2018	c.250−?_396+?del	Exon 5 del	Microarray-CGH, qPCR	No	–	RCC
(11)	Iwabuchi et al., 2018	c.1063−?_1176+?del	Exon 10 del	Not mentioned	No	–	LC, PTX, renal cysts
		c.1539−?_c.1740+?del	Exon 14 del	Not mentioned	No	–	LC, PTX, skin trichodiscoma
(12)	Enomoto et al., 2020	c. −?_−227−?del	Exon 1 del	qPCR	No	–	LC, SL, RCC
(13)	This study	c.1063–1446_1740+3410del	Exon 10–14 del	NGS, MLPA	Yes, flanked by Alu-Sg and Alu-Sx1	8,384	PTX, LC, SL

*PTX, pneumothorax; FF, fibrofolliculomas; LC, lung cysts; RCC, renal cell carcinoma; SL, skin lesion (not histopathologically confirmed).*

*Reference genome, GRCh37/hg19; accession number: FLCN, NM_144997.7.*

All the breakpoints were finally defined by long-range PCR followed by DNA sequencing. For deletions detected by qPCR and MLPA, multiple pairs of primers flanking the deleted exon(s) were designed for PCR reaction. While in our present study, as we optimized the NGS method by including the 5′ flank and 3′ flank sequence, the boundaries of the deletions were further minimized, only one pair of primer set was required to successfully define the breakpoints.

Although our present data shows an equal effectiveness between MLPA and NGS methods in detecting exon deletions (Figure 3A). We also observed that MLPA could exhibit false positive results when the probe cannot combine with DNA at a locus with small indel (Vorstman et al., 2006; Lim et al., 2010; Zhang et al., 2016), resulting in a decreased probe hybridization and ligation with the target DNA sequences, and consequently loss of amplification (Figure 3C). Due to the occasionally false positives, we suggest that breakpoint analysis should be conducted in patients with positive MLPA results to confirm the deletions. In addition, according to the study of other researchers, partial exonic deletions could escape MLPA detection, while NGS method has the capacity to detect them (Liu et al., 2018).

The incidence of large intragenic deletions is high, so normal screening must take this into consideration, this makes the genetic diagnostic procedure complex, as Sanger sequencing and MLPA must be both performed. Targeted next-generation sequencing method could identify the full spectrum of *FLCN* gene mutations, as well as determining deletion junctions in a single experiment, and has proven to be a highly sensitive, accurate and cost-saving tool for detecting single nucleotide variations, indels and CNVs in the previous and present studies (Zhang et al., 2016).

Considering all of the above, we highly recommend targeted NGS technique, as a diagnostic application, be widely used for clinical molecular diagnosis in suspect BHDS patients.

Here we reported a Chinese BHDS family with a novel *FLCN* intragenic deletion identified by NGS. The affected family members should be followed up in the following years in case of renal cancer and other symptoms. Our report expands the mutation spectrum of the disease-causing gene.

## Data Availability Statement

The datasets generated for this study can be found in online repositories. The names of the repository/repositories and accession number(s) can be found below: NCBI, FLCN: NM_144997.7 (https://www.ncbi.nlm.nih.gov/nuccore/NM_144997.7), MW600551, and MW600552.

## Ethics Statement

The studies involving human participants were reviewed and approved by Ethical Committees of Taizhou Hospital of Zhejiang Province. The patients/participants provided their written informed consent to participate in this study.

## Author Contributions

MC, XZ, QG, CZ, LY, and DM conceived and designed the study, contributed to data analysis and interpretation, manuscript drafting, and critical review for intellectual content and final approval of the manuscript. MC, BC, MY, CZ, and DM co-designed experiments, contributed to collection of the clinical data, manuscript editing and discussed analyses, interpretation, and presentation. XZ, LF, SCh, AK, and SCe performed the experiments, contributed to data analysis, and manuscript editing. All authors read and approved the final manuscript.

## Conflict of Interest

The authors declare that they have no known competing financial interests or personal relationships that could have appeared to influence the work reported in this paper.

## Footnotes

1 https://databases.lovd.nl/shared/genes/FLCN

2 https://databases.lovd.nl/shared/genes/FLCN

3 https://www.ncbi.nlm.nih.gov/clinvar/
