# Supplementary material for: A Novel FLCN Intragenic Deletion Identified by NGS in a BHDS Family and Literature Review
Source: Front Genet. 2021 Apr 1;12:636900. doi: 10.3389/fgene.2021.636900 (PMC8078137; doi:10.3389/fgene.2021.636900)
Supplement: Supplementary file 1 [file Data_Sheet_1.docx]

**Methods**

**Ethics statement, DNA isolation and Sanger sequencing**

Blood samples were collected from the proband (II-2), Ⅰ-1, Ⅱ-1, Ⅲ-1, Ⅲ-2 and Ⅲ-3 respectively (Fig. 1A), with written informed consent from each participant. Genomic DNA was extracted from peripheral blood samples of collected family members using a Qiagen DNA Mini blood Kit (Qiagen, Hilden, Germany) according to manufacturer instruction. Mutation analysis was performed by direct DNA sequencing of all coding exons and all intron-exon boundaries including at least 50 intronic nucleotides of *FLCN*. The primers were designed using the online software Primer3 (Table S1). Sequencing results were compared with the reference DNA sequences derived from GenBank (accession number: *FLCN*: NM_144997.7) and then reviewed manually.

**HaloPlex design, target enrichment, and next generation sequencing**

A custom Haloplex panel was designed using Agilent’s online SureDesign tool (https://earray.chem.agilent.com/suredesign/index.htm). In all, 68 targeted region including the whole gene sequences of *FLCN*, *FNIP1*, *FNIP2* covered the 5’ flank and 3’ flank sequence, and all coding exons, intron-exon boundaries including 50 intronic nucleotides and 5’ Untranslated Regions (UTR), 3′ UTR of *TSC1*, *TSC2* were captured for enrichment. The target regions were captured using the Agilent HaloPlex Target Enrichment System Kits for Illumina Sequencing (Custom Panel Tier 1, ILM, 48 reactions; Agilent Technologies, Inc. Santa Clare, CA) following Agilent protocols. Afterwards, equimolar amounts of differentially indexed samples were pooled before pair-ended sequencing 300 bp on the Illumina MiSeq platform (Illumina Inc., San Diego, CA, USA), and the mean read depth within the regions of interest was >100 reads per base (Table S2).

**Copy-number analysis (CNV) and breakpoint analysis for NGS data**

CNVs were identified using a depth-based method. Coverage statistics were generated using GATK Depth of Coverage tool version 3.1. We normalized depth in each sample by dividing the average depth of each exon by the total number of on-target mapped reads for that sample (normalized depth = $\frac{\text{the average depth of each exon}}{\text{the total number of on-target mapped reads for that sample}}$) (1). To identify copy number variations at individual exons, we compared the normalized depth of each exon of the individual patient with that of all testing patients in the same targeted NGS experiment. Coverage data and bars/plots graph were produced using Prism 6. Exons with a normalized depth ratio below/above 0.7/1.3 of the mean in controls were classified as heterozygously deleted/duplicated. A 20-bp interval was used for approximate breakpoint determination (Fig. S2).

**Multiplex Ligation-Dependent Probe Amplification (MLPA) and precise breakpoint analysis**

MLPA assay was performed to detect large intragenic deletions/duplications using the commercial kit P256-B1 FLCN (MRCHolland, Netherlands, http://www.mlpa.com). MLPA reactions were performed by following the manufacturer’s instructions. The polymerase chain reaction (PCR) products were analyzed on an ABI 3130 Genetic Analyzer (Applied Biosystems). Data were analyzed using the coffalyser software (MRC-Holland).

The junction fragments adjacent to the deleted regions were amplified using the PCR Amplification Kit (Takara, China) with specially designed primers (Primer F: GGAGTTGTGGAGGTATAAAGAAGGA; Primer R: TTGGGGGTATTAGCCATAGGA). PCR products were separated by 2% agarose gel electrophoresis. Bidirectional sequencing was performed. Deletions were named under the HGVS nomenclature guideline and numbered by referring to the FLCN cDNA reference sequence (NM_144997.6, +1 = A of ATG) obtained from the NCBI database.

**Linkage and haplotype analysis**

Four short tandem repeat (STR) polymorphic markers D17S122, D17S740, D17S2196 and D17S1824 flanking *FLCN* region (chr17:17,115,527-17,140,502) were detected to determine the haplotype of each patient. The markers were amplified by PCR. Markers were genotyped in all family members obtained, and linkage analysis was performed with ABI Prism 3130 Genetic Analyzer (Applied Biosystems, Foster City, CA, USA) and the length of each allele was determined by the GeneMapper software (Applied Biosystems, Foster City, CA). To minimize the number of crossovers in the family, haplotypes were constructed and assigned.

**Data collection and Analysis**

To evaluate the deletion-associated phenotype of BHD syndrome, relevant studies were searched from the database PubMed and four previous studies were selected (2-5). The clinical manifestations and *FLCN* gene mutations were obtained. The number of patients with the presence of lung cysts, skin lesions, renal tumors and history of pneumothorax were counted and compared between *FLCN* intragenic deletions and other *FLCN* mutations (nonsense, indel and splice site). Statistical significance was set at P < 0.05 and determined by the chi-square test. Data analysis was performed using R for Windows version 4.0.2.

**Supplementary reference:**

1. Zhang X, Ma D, Zou W, Ding Y, Zhu C, Min H, et al. A rapid NGS strategy for comprehensive molecular diagnosis of Birt-Hogg-Dube syndrome in patients with primary spontaneous pneumothorax. *Respir Res* (2016) 17(1):64. Epub 2016/05/28. doi: 10.1186/s12931-016-0377-9. PubMed PMID: 27229674; PubMed Central PMCID: PMCPMC4882857.

2. Toro JR, Wei MH, Glenn GM, Weinreich M, Toure O, Vocke C, et al. BHD mutations, clinical and molecular genetic investigations of Birt-Hogg-Dube syndrome: a new series of 50 families and a review of published reports. *J Med Genet* (2008) 45(6):321-31. Epub 2008/02/01. doi: 10.1136/jmg.2007.054304. PubMed PMID: 18234728; PubMed Central PMCID: PMCPMC2564862.

3. Kunogi M, Kurihara M, Ikegami TS, Kobayashi T, Shindo N, Kumasaka T, et al. Clinical and genetic spectrum of Birt-Hogg-Dube syndrome patients in whom pneumothorax and/or multiple lung cysts are the presenting feature. *J Med Genet* (2010) 47(4):281-7. Epub 2010/04/24. doi: 10.1136/jmg.2009.070565. PubMed PMID: 20413710; PubMed Central PMCID: PMCPMC2981024.

4. Liu Y, Xu Z, Feng R, Zhan Y, Wang J, Li G, et al. Clinical and genetic characteristics of chinese patients with Birt-Hogg-Dube syndrome. *Orphanet J Rare Dis* (2017) 12(1):104. Epub 2017/06/01. doi: 10.1186/s13023-017-0656-7. PubMed PMID: 28558743; PubMed Central PMCID: PMCPMC5450333.

5. McDermott C, Cullen J. Familial Birt-Hogg-Dube syndrome. *QJM* (2018) 111(8):565-6. Epub 2018/04/19. doi: 10.1093/qjmed/hcy079. PubMed PMID: 29669049.

Table S1 Primers designed to perform direct sequencing for each exon of *FLCN*

| **Exon** | **F** | **R** |
| --- | --- | --- |
| Exon 1 | CAACGAAAACTCGGACATGC | CAGTGTTGGGTGGTGGTACG |
| Exon 2 | ATCAGGGGAAGAGAAATAAGGTTC | CATGCTACGAAGGCCTCTAATC |
| Exon 3 | CTGCCAAAGCCGCTAACTCT | CCAGCCCAGCTAAGGTTCTCT |
| Exon 4 | GGGAGGTTTCATGGAGTCAA | CTCTCAGGTCCTCCTGTCCAT |
| Exon 5 | CCCTGCTTCCCAACTAACAG | GCAAGTCCAACATGACTCCTC |
| Exon 6 | TCAGCACAGAGCGGCTCATG | GAAGAGGCTTTGATTTGGTGTCAC |
| Exon 7 | GCATGGAGAGAGTATAGTGGGACT | GCCAACCAATGTATCGTGACT |
| Exon 8 | AGGGAACCACTGCCCTTCAT | TCAGGTTTGCTTTTTCCTTTGG |
| Exon 9 | CCATGAAGTATCTTGGGCTGA | GAGGCTGTCAGTCACTTCCTG |
| Exon 10 | GTCTTTCTCCTGAGCCCTGTC | CAGTGGAGACCGTGTGGTG |
| Exon 11 | GGTTTGGGTAGTAGAGCATGGA | CAGAGATCTGGTTCCACTTTGG |
| Exon 12-exon 13 | CAGCTCCAGGTTTTCTCCAGG | CACGGTGGGCTAGCGCAG |
| Exon 14 | ACCAGGGCTCGAGGGATTG | TGTCTTTAGGCAGGTGTGTGTGA |

Table S2 Mean depth of the targeted genes

| **Gene** | **Mean depth** | **Standard error** |
| --- | --- | --- |
| FNIP2 | 310.68 | 87.70 |
| FNIP1 | 197.46 | 55.42 |
| TSC1 | 119.10 | 34.53 |
| TSC2 | 172.74 | 54.41 |
| FLCN | 363.25 | 112.82 |

Table S3 A normalized depth ratio for each exon in FLCN gene in the proband (II-2).

| **Exon** | **Normalized depth ratio** |
| --- | --- |
| Exon 1 | 0.918987547 |
| Exon 2 | 0.980640064 |
| Exon 3 | 0.983507099 |
| Exon 4 | 1.030101401 |
| Exon 5 | 0.975841841 |
| Exon 6 | 0.97369602 |
| Exon 7 | 0.901546902 |
| Exon 8 | 0.94421033 |
| Exon 9 | 1.259076536 |
| Exon 10 | 0.488828295 |
| Exon 11 | 0.478292688 |
| Exon 12 | 0.499313492 |
| Exon 13 | 0.607090051 |
| Exon 14 | 0.530626364 |

Table S4 A comparison of clinical manifestation between deletion mutations and other mutations in BHD patients.

| **Mutation type** | **No. clinically affected** | **No. with LC** | **No. with PTX** | **No. with SL** | **No. with RT** |
| --- | --- | --- | --- | --- | --- |
| *FLCN* nonsense, indel and splice site mutations | 145 | 130 | 82 | 84 | 33 |
| *FLCN* intragenic deletions/duplications | 83 | 68 | 45 | 31 | 9 |
| P value |  | 0.7325 | 0.9464 | 0.1027 | 0.0883 |

LC: lung cysts, PTX: pneumothorax, SL: skin lesions, RT: renal tumors

Fig. S1 Detection of copy number variants. Representation of the normalized depth ratio of each exon in *FLCN* gene. Exons with a normalized coverage ratio below 0.7 were classified as heterozygously deleted.

**

**

Fig. S2 A visualized flow chart of deletion detection and precise breakpoints determination.

**
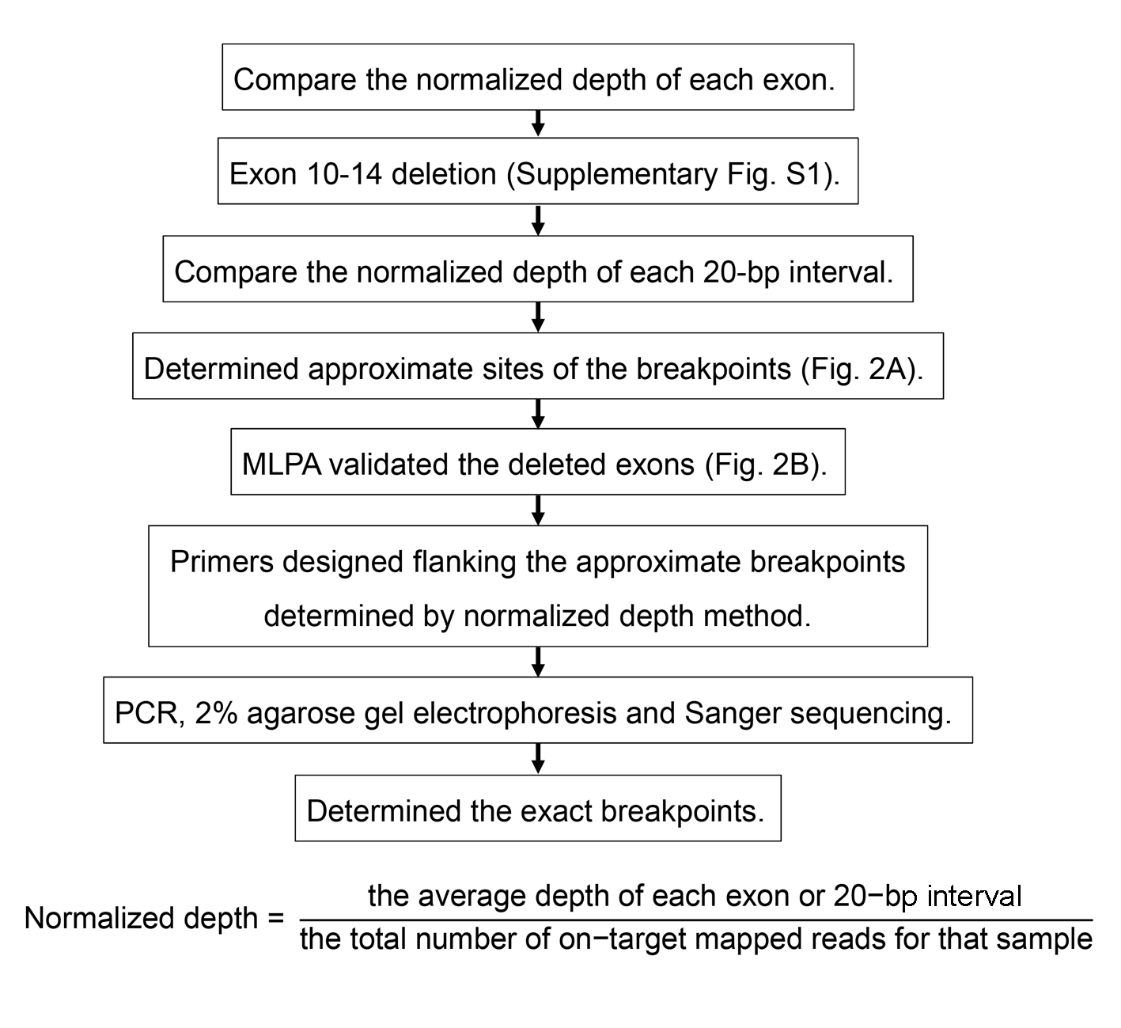
**
